# Clusterin regulates macrophage expansion, polarization and phagocytic activity in response to inflammation in the kidneys

**DOI:** 10.1111/imcb.12405

**Published:** 2020-10-16

**Authors:** Xiaodong Weng, Haimei Zhao, Qiunong Guan, Ganggang Shi, Shijian Feng, Martin E Gleave, Christopher CY Nguan, Caigan Du

**Affiliations:** ^1^ Department of Urologic Sciences The University of British Columbia Vancouver BC V5Z 1M9 Canada; ^2^ Department of Urology Renmin Hospital of Wuhan University Wuhan Hubei 430060 China; ^3^ College of Traditional Chinese Medicine Jiangxi University of Traditional Chinese Medicine Nanchang Jiangxi Province 330004 China; ^4^ Department of Colorectal Surgery The Second Hospital of Tianjin Medical University Tianjin 300211 China

**Keywords:** clusterin, macrophage activation, macrophage phenotype, kidney injury, kidney repair

## Abstract

Clusterin (CLU) is a multifunctional protein localized extracellularly and intracellularly. Although CLU‐knockout (KO) mice are more susceptible to renal ischemia‐reperfusion injury (IRI), the mechanisms underlying the actions of CLU in IRI are not fully understood. Macrophages are key regulators of IRI severity and tissue repair. Therefore, we investigated the role of CLU in macrophage polarization and phagocytosis. Renal IRI was induced in wild‐type (WT) or CLU‐KO C57BL/6 mice by clamping the renal pedicles for 30 min at 32°C. Peritoneal macrophages were activated via an intraperitoneal injection of lipopolysaccharide (LPS). Renal tissue damage was examined using histology, whereas leukocyte phenotypes were assessed using flow cytometry and immunohistochemistry. We found that monocytes/macrophages expressed the CLU protein that was upregulated by hypoxia. The percentages of macrophages (F4/80^+^, CD11b^+^ or MAC3^+^) infiltrating the kidneys of WT mice were significantly less than those in CLU‐KO mice after IRI. The M1/M2 phenotype ratio of the macrophages in WT kidneys decreased at day 7 post‐IRI when the injury was repaired, whereas that in KO kidneys increased consistently as tissue injury persisted. In response to LPS stimulation, WT mice produced fewer M1 macrophages, but not M2, than the control did. Phagocytosis was stimulated by CLU expression in macrophages compared with the CLU null controls and by the exogenous CLU protein. In conclusion, CLU suppresses macrophage infiltration and proinflammatory M1 polarization during the recovery period following IRI, and enhances phagocytic activity, which may be partly responsible for tissue repair in the kidneys of WT mice after injury.

## Graphical abstract

In this study, we found that macrophages expressed clusterin, and knockout of clusterin expression disrupted the decrease of infiltrating macrophages and M2 polarization during the late stage of recovery after ischemia‐reperfusion injury in the kidney.

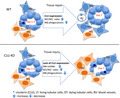

## INTRODUCTION

Ischemia‐reperfusion injury (IRI) is one of the major causes of acute kidney injury,^1^ which occurs in more than 50% of patients admitted to the intensive care unit.^2^ It is correlated with higher morbidity and mortality, as well as higher risk of developing chronic kidney disease.^3^, ^4^ Renal IRI is typically characterized by the activation of inflammation, such as the infiltration of neutrophils, macrophages (Mφ), dendritic cells, natural killer cells, CD4^+^ and CD8^+^ T cells, regulatory T cells, natural killer T cells and B cells, along with the apoptosis of tubular epithelial cells (TECs) and endothelial cells in the kidney.^5^, ^6^ Among these infiltrating leukocytes, the Mφ play a key role in the damage and repair, inflammation and fibrosis in renal tissues.^7^

Mφ, a common phagocytic leukocyte, are involved in tissue homeostasis and inflammation.^8^, ^9^ Several phenotypes of Mφ populations have been proposed,^10^, ^11^ of which the most important and well‐characterized are the M1 and M2. The M1 Mφ mediate the inflammatory killing of infective pathogens and tumor cells, whereas the M2 Mφ are the anti‐inflammatory cells that promote cell proliferation and tissue healing or repair.^12^, ^13^ In the kidneys with IRI, the proinflammatory M1 phenotype has been demonstrated to be predominant in the early phase and mediates tissue damage. By contrast, the M2 phenotype is predominant in the late recovery phase and contributes to tissue repair.^14^, ^15^ Both the M1 and M2 Mφ coexist in the damaged kidney tissues, albeit at a different disease stages. In addition, the M1 Mφ may transform into M2 Mφ within the kidney between the early stage of injury and the postinjury recovery phase.^7^, ^15^, ^16^ Therefore, the M1 and M2 Mφ are not been considered to be completely independent types of cells, but rather they are two distinguishable phenotypes of Mφ population involved in the pathogenesis of renal injury.^7^, ^14^, ^15^, ^16^ This suggests that the phenotypes of Mφ may dynamically change depending on the stage of renal IRI, that is, injury or recovery. However, the mechanisms underlying the plasticity and the activation of Mφ during kidney injury and repair are not fully understood.

Clusterin (CLU) is a 75–80‐kDa multifunctional heterodimer glycoprotein that has been first reported in 1983 as an adhesion glycoprotein in goat semen.^17^ CLU has been associated with various physiological processes and pathological states, such as cancer progression, neurodegenerative disorders, aging, immune diseases, dry eye disease and metabolic/cardiovascular diseases.^18^, ^19^ In addition, CLU is involved in a variety of kidney disorders, such as IRI,^20^ unilateral ureteral obstruction,^21^ rejected renal allografts,^22^ and intrinsic kidney disease.^23^, ^24^ Furthermore, studies on CLU‐knockout (KO) mice have found that (i) CLU deficiency accelerated renal fibrosis in response to IRI and unilateral ureteral obstruction^21^, ^25^ (ii) in aging mice, CLU deficiency resulted in the development of progressive glomerulopathy that has been associated with glomerular antibody deposition^26^ and (iii) CLU‐KO mice exhibited greater severity of renal IRI, as well as the impairment of renal repair after IRI.^20^, ^27^ Interestingly, among the different types of infiltrating leukocytes, only the numbers of Mφ and CD8^+^ T cells were increased in the kidneys of CLU‐KO mice compared with the CLU‐expressing, wild‐type (WT) mice after 30 days of IRI.^25^ However, whether CLU regulates the Mφ activation or the polarization in the kidneys with IRI during the early stage of tissue damage and the late stage of repair remains uninvestigated. Therefore, in this study, we investigated the effects of CLU on Mφ polarization and activation using the CLU‐KO mice as the CLU null control in a renal IRI model. The novel finding of this study was that the lack of CLU expression in Mφ impaired the development of M2 phenotype during the recovery phase in the kidney after IRI.

## RESULTS

### CLU is associated with fewer Mφ in kidneys after IRI

**Figure 1:**
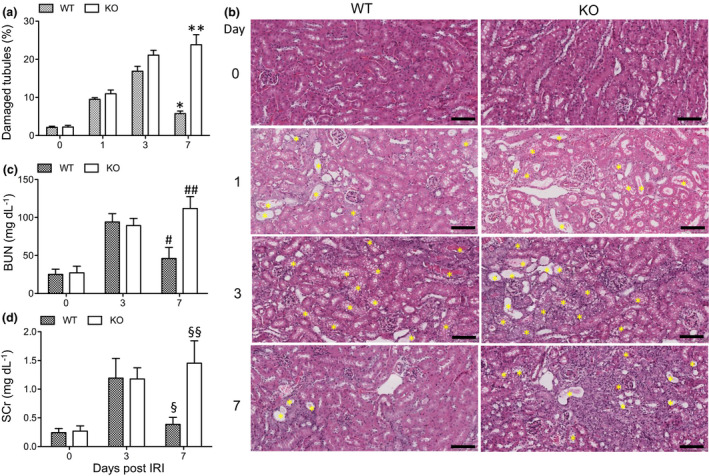
Evaluation of CLU expression in kidney tissues following renal IRI. Kidney sections of WT and CLU‐KO mice at 0, 1, 3 and 7 days following IRI were stained with hematoxylin and eosin. **(a)** The number of damaged kidney tubules in the cortex were blindly counted as stated in the “Methods” section. Data are representative of three independent experiments and presented as the mean ± s.e.m. (*n* = 3 per time point). *P = 0.0016 (day 3 *versus* 7 in WT, two‐tailed *t*‐test); **P = 0.0027 (WT *versus* KO at day 7, two‐tailed *t*‐test). **(b)** Representative images of the kidney cortex from three independent experiments. Yellow asterisks indicate damaged tubules (i.e. tubular vacuolation, necrotic tubules, intratubular cast formation and tubular dilatation). Scale bar: 100 µm. **(c)** BUN levels and **(d)** SCr levels after the removal of nonischemic right kidney. Data are representative of three independent experiments and presented as the mean ± s.d. (*n* = 4 per time point per group). **(c)** ^#^ *P* = 0.0019 (day 3 *versus* 7 in WT); ^##^ *P* = 0.0008 (WT *versus* KO at day 7); **(d)** ^§^ *P* = 0.0045 (day 3 *versus* 7 in WT); ^§§^ *P* = 0.0020 (WT *versus* KO at day 7) using the two‐tailed *t*‐test. BUN, blood urea nitrogen; CLU, clusterin; IRI, ischemic‐reperfusion injury; KO, clusterin knockout; SCr, serum creatinine; WT, wild type.

As shown in Figure 1a, b, renal tubular damage, including tubular necrosis, tubular dilatation and tubular vacuolization, and inflammatory cell infiltration were observed after renal IRI. The renal tubular damage in both WT and CLU‐KO mice was significantly induced at day 1 with no significant difference (WT, 9.5 ± 0.75%; KO, 10.97 ± 1.72%; *P* = 0.2463, *n* = 3). The damage increased and reached the maximum at day 3, with the control showing greater damage than the WT group (WT, 16.9 ± 2.25%; KO, 21.1 ± 2.23%; *P* = 0.0833, *n* = 3). At day 7, tubular damage in WT mice decreased to 5.77 ± 1.17% (repaired) (day 3 *versus* 7, *P* = 0.0016), whereas in KO mice, it progressively increased to 23.83 ± 4.59% (WT *versus* KO, *P* = 0.0027, *n* = 3) (Figure 1a). These results confirmed our previous observation^27^; both indicated that CLU mediated the renal tissue repair after IRI. The tissue repair of the kidney at day 7 in the WT group was consistent with its functional recovery, as indicated by decreases in blood urea nitrogen (day 3, 94.0 ± 11.0 mg dL^−1^; day 7, 46.03 ± 14.54 mg dL^−1^; *P* = 0.0019, *n* = 4) and serum creatinine levels (day 3, 1.19 ± 0.34 mg dL^−1^; day 7, 0.39 ± 0.12 mg dL^−1^; *P* = 0.0045, *n* = 4) (Figure 1c, d). These functional parameters did not decrease in the KO group (Figure 1c, d), which was consistent with the absence of tissue repair at day 7 after IRI.

**Figure 2:**
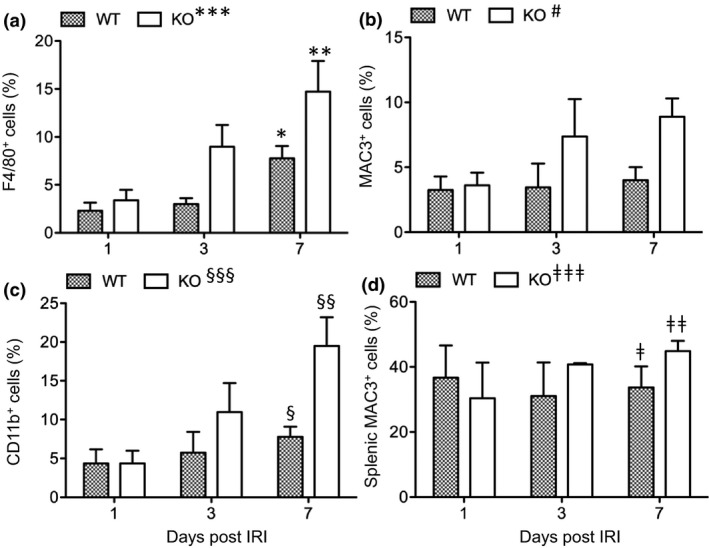
CLU expression is associated with decreased macrophage expansion in the kidney following renal IRI. Macrophages were identified using the positively stained F4/80, MAC3 or CD11b cells and were counted by FACS analysis. **(a)** F4/80^+^ cells in the kidneys of the WT and CLU‐KO groups following IRI from day 1 to day 7. *P = 0.0014 (WT, one‐way ANOVA); **P = 0.0114 (KO, one‐way ANOVA); ***P = 0.0291 (WT *versus* KO, two‐way ANOVA). **(b)** MAC3^+^ cells. ^#^ *P* = 0.0434 (WT *versus* KO, two‐way ANOVA). **(c)** CD11b^+^ cells. ^§^ *P* = 0.5201 (WT, one‐way ANOVA); ^§§^ *P* = 0.0406 (KO, one‐way ANOVA); ^§§§^ *P* = 0.0230 (WT *versus* KO, two‐way ANOVA). **(d)** MAC3^+^ in the spleens of the WT and CLU‐KO groups following IRI from day 1 to day 7. ^ǂ^ *P* = 0.9093 (WT, one‐way ANOVA); ^ǂǂ^ *P* = 0.3412 (KO, one‐way ANOVA); ^ǂǂǂ^ *P* = 0.4669 (WT *versus* KO, two‐way ANOVA). Data in **a** (*n* = 7 at each time point), **b** (*n* = 3 at each time point), **c** (*n* = 3 at each time point) and **d** (*n* = 3 per time point) are representative of two independent experiments and presented as the mean ± s.e.m. CLU, clusterin; FACS, fluorescence‐activated cell sorting; IRI, ischemic‐reperfusion injury; KO, clusterin knockout; WT, wild type.

To elucidate the role of CLU in kidney repair, its effect on Mφ activation in the kidneys after IRI was examined. The numbers of Mφ in the kidneys of both groups were increased regardless of the marker. Particularly, F4/80^+^ cells increased (day 1, 2.32 ± 2.2%; day 7, 7.76 ± 3.43%; *P* = 0.0014, *n* = 7) in both the WT and the control groups (day 1, 3.39 ± 2.89%; day 7, 14.72 ± 8.45%; *P* = 0.0114, *n* = 7) (Figure 2a). Similar data were seen in MAC3^+^ (Figure 2b) and CD11b^+^ cells (Figure 2c). Importantly, there were fewer Mφ in the WT kidneys from day 1 to day 7, particularly at day 7 when the kidney injury was repaired, than in the CLU‐KO control (Figure 1). As shown in Figure 2a, b, c, significantly fewer F4/80^+^ cells (*P* = 0.0030, *n* = 7), MAC3^+^ cells (*P* = 0.0434, *n* = 3) and CD11b^+^ cells (*P* = 0.0230, *n* = 3) were observed in the WT group than in the control. As shown in Figure 2d, the percentages of splenic MAC3^+^ cells in either group remained statistically unchanged from day 1 to day 7 after renal IRI and were not significantly different (*P* = 0.4669, *n* = 3). Taken together, these data suggested that CLU suppressed the expansion (infiltration and/or proliferation) of Mφ after renal IRI.

**Figure 3:**
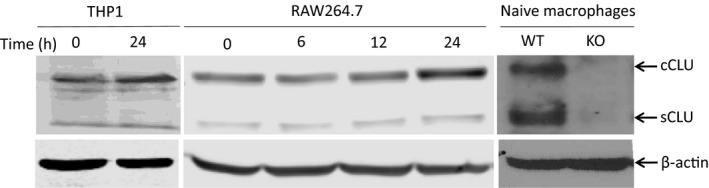
CLU expression in macrophages under normoxia or hypoxia. Cellular protein extracts were harvested from THP1 cells after exposure to hypoxia (1% O_2_) for 0 or 24 h; from RAW264.7 cells after hypoxia for 0 to 24 h and from peritoneal macrophages of WT and CLU‐KO mice. The cytoplasmic CLU (cCLU or presecreted CLU) and mature or secreted CLU (sCLU) proteins were detected with anti‐CLU antibody using the western blot (top panel). The level of β‐actin was used for normalization (bottom panel). Data are representations of three independent examinations. CLU, clusterin; cCLU, cytoplasmic clusterin; KO, clusterin knockout; sCLU, mature or secreted clusterin; WT, wild type.

### CLU levels in Mφ and monocytes

The abundances of CLU proteins were observed in cultured human THP1 monocytes, mouse RAW264.7 Mφ and primary peritoneal Mφ using the western blotting (Figure 3). Under normoxic conditions, the THP1, RAW264.7 and primary peritoneal Mφ from the WT mice produced presecreted or cytoplasmic (~58 kDa) CLU and the mature or secreted CLU (~40 kDa). CLU levels were significantly upregulated in both THP1 and RAW264.7 cells after 24 h of hypoxia (1% O_2_). The presence of CLU proteins in these monocytes and Mφ was further confirmed by the lack of CLU proteins in the Mφ of CLU‐KO mice.

### CLU is associated with decreased M1/M2 ratio of Mφ in the repair phase after IRI

**Figure 4:**
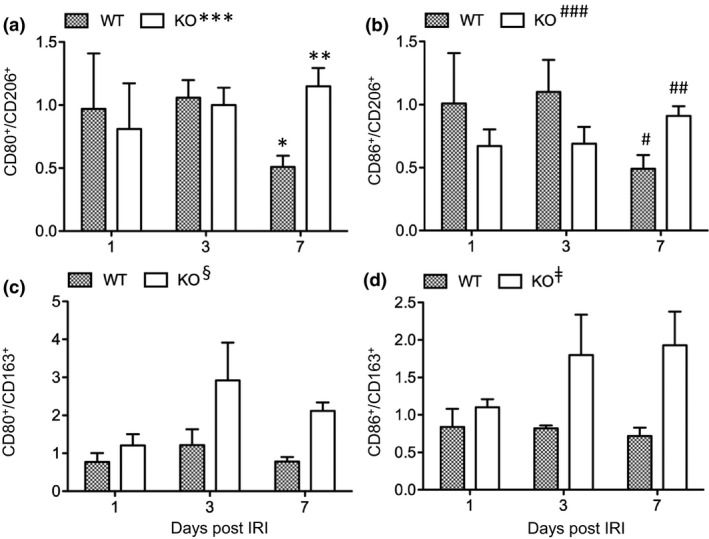
CLU expression is associated with decreased M1/M2 ratios during kidney repair following renal IRI. The macrophage population was identified using F4/80^+^ gating, and the numbers of M1 (CD80^+^ and CD86^+^) and M2 (CD206^+^ and CD163^+^) macrophages were counted by FACS analysis. **(a)** CD80^+^/CD206^+^ ratios in the kidneys. *P = 0.0093 (day 3 *versus* 7 in WT); **P = 0.5730 (day 3 *versus* 7 in KO); ***P = 0.0021 (WT *versus* KO at day 7). **(b)** CD86^+^/CD206^+^ ratios. ^#^ *P* = 0.0212 (day 3 *versus* 7 in WT); ^##^ *P* = 0.1618 (day 3 *versus* 7 in KO); ^###^ *P* = 0.0037 (WT *versus* KO at day 7). **(c)** CD80^+^/CD163^+^ ratios. ^§^ *P* < 0.0001 (WT *versus* KO at day 7). **(d)** CD86^+^/CD163^+^ ratios. ^ǂ^ *P* = 0.0133 (WT *versus* KO at day 7). Data are representative of two independent experiments and presented as the mean ± s.e.m. (at days 1 and 3, *n* = 3 per group; and day 7, *n* = 8 per group). The difference between groups was analyzed by using the two‐tailed *t*‐test. CLU, clusterin; FACS, fluorescence‐activated cell sorting; IRI, ischemic‐reperfusion injury; KO, clusterin knockout; WT, wild type.

To investigate the role of CLU levels in Mφ cell activation and function as defined by the M1 or M2 phenotype during renal IRI and repair, the expression of the M1 marker (CD80 or CD86) and the M2 marker (CD206 or CD163) on primarily gated F4/80^+^ Mφ (M1, F4/80^+^CD80^+^ and F4/80^+^CD86^+^; M2, F4/80^+^CD206^+^ and F4/80^+^CD163^+^) was evaluated by fluorescence‐activated cell sorting (FACS) analysis. The M1/M2 ratios of F4/80^+^ Mφ were represented using the CD80^+^/CD206^+^, CD86^+^/CD206^+^, CD80^+^/CD163^+^ and CD86^+^/CD163^+^. The changes of the M1/M2 ratios at different days after IRI were similar between CD80^+^/CD206^+^ and CD86^+^/CD206^+^ (Figure 4a, b) and between CD80^+^/CD163^+^ and CD86^+^/CD163^+^ (Figure 4c, d). The M1/M2 ratios of CD80^+^/CD206^+^ and CD86^+^/CD206^+^ in the WT group at day 7 (*n* = 8) were significantly decreased compared with those at day 3 (*n* = 3; CD80^+^/CD206^+^, *P* = 0.0093; CD86^+^/CD206^+^, *P* = 0.0212). In the control group, the ratios of these cells at day 7 (*n* = 8) were not significantly different from those at day 3 (*n* = 3) (CD80^+^/CD206^+^, *P* = 0.5730; CD86^+^/CD206^+^, *P* = 0.1618). At day 7, the CD80^+^/CD206^+^ and CD86^+^/CD206^+^ ratios were significantly lower in the WT than in the control (CD80^+^/CD206^+^, *P* = 0.0071; CD86^+^/CD206^+^, *P* = 0.0037, *n* = 8). As shown in Figure 4c, d, the M1/M2 ratios of CD80^+^/CD163^+^ and CD86^+^/CD163^+^ were unchanged at day 7 compared with those at day 3 (CD80^+^/CD163^+^, *P* = 0.1729; CD86^+^/CD163^+^, *P* = 0.5946) in the WT group but were significantly less than those in the control at day 7 (CD80^+^/CD163^+^, *P* < 0.0001; CD86^+^/CD163^+^, *P* = 0.0133, *n* = 8). Overall, these data suggested that the renal repair at day 7 in the WT group was positively correlated with decreased M1/M2 ratios in the renal F4/80^+^ Mφ compared with the control.

**Figure 5:**
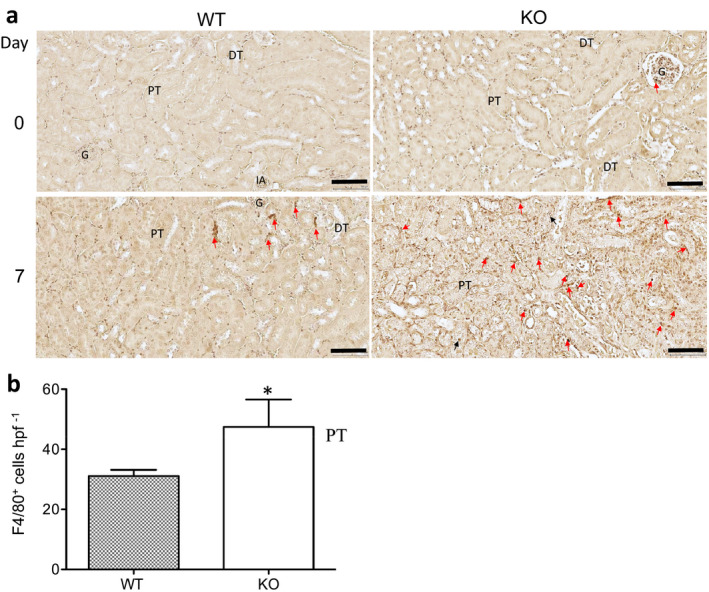
CLU expression is associated decreased macrophage expansion during kidney repair at day 7 following renal IRI. The macrophages in the kidney sections of WT and CLU‐KO mice were harvested at day 7 following IRI and were identified using the immunohistochemistry. **(a)** Representative images of three independent experiments. F4/80^+^ cells (dark brown) in the tubulointerstitial area, perivascular space and injured tubular epithelium of the renal cortical and medullary junction. Red arrows indicate positively stained cells; Black arrows correspond to false‐positive staining. Scale bar: 100 µm. **(b)** The numbers of F4/80^+^ cells. Data are representative of three independent experiments and presented as the mean ± s.d. (*n* = 3). *P = 0.0386 (WT *versus* KO, two‐tailed *t*‐test). CLU, clusterin; DT, distal convoluted tubule; G, glomerulus; IA, interlobular artery; KO, clusterin knockout; PT, proximal convoluted tubule; WT, wild type.

Nitric oxide synthase 2 (NOS 2) is a biomarker of M1 Mφ, whereas arginase 1 (ARG 1) is a biomarker of M2 Mφ.^28^ We also examined the expression of F4/80, NOS 2 and ARG 1 in the kidney sections of WT and CLU‐KO groups at day 7 using immunohistochemistry. As shown in Figure 5a, the F4/80^+^ cells were mainly localized in the interstitial and perivascular areas, and there were a small number of injured TEC that were false positive and thus were not counted. There were fewer F4/80^+^ Mφ (31.11 ± 2.04 cells hpf^−1^) in the WT group than in CLU‐KO kidneys (47.48 ± 9.12 cells hpf^−1^, *P* = 0.0386, *n* = 3) (Figure 5b), which were consistent with FACS analysis of the numbers of F4/80^+^ Mφ (Figure 2a).

**Figure 6:**
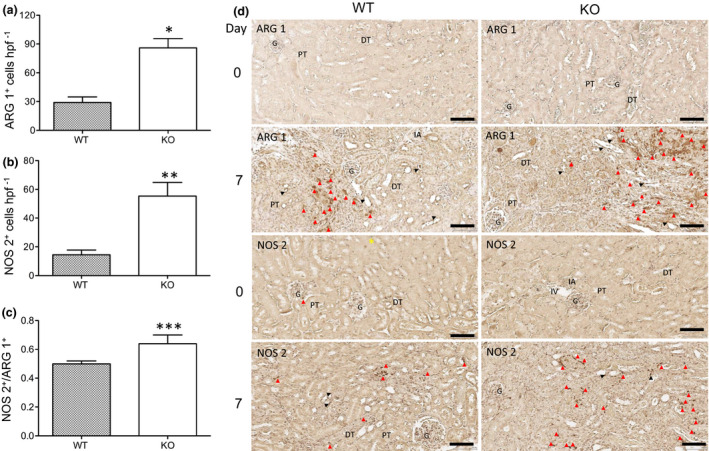
CLU expression is associated with decreased ratio of NOS 2^+^ (M1 marker)/ARG 1^+^ (M2 marker) cells at day 7 following renal IRI. ARG 1^+^ and NOS 2^+^ cells were identified by immunohistochemistry as described in the “Methods” section. **(a)** The numbers of ARG 1^+^ cells. *P = 0.0010 (WT *versus* KO, two‐tailed *t*‐test). **(b)** The numbers of NOS 2^+^ cells. **P = 0.0021 (WT *versus* KO, two‐tailed *t*‐test). **(c)** The difference in the M1/M2 ratio between the WT and CLU‐KO groups was confirmed by the NOS 2^+^/ARG 1^+^ ratio in each kidney. ***P = 0.0179 (WT *versus* KO, two‐tailed *t*‐test). Data in **a**, **b** and **c** are representative of three independent experiments and presented as the mean ± s.d. (*n* = 3). **(d)** Representative images of three independent experiments. ARG 1^+^ (top 2) and NOS 2^+^ (bottom 2) cells (dark brown) in the tubulointerstitial area, the perivascular space and injured tubular epithelium of the renal cortical and medullary junction. Red arrowheads represent positively stained cells; Black arrowheads correspond to false‐positive staining. Scale bar: 100 µm. ARG 1; arginase 1; CLU, clusterin; DT, distal convoluted tubule; G, glomerulus; IA, interlobular artery; KO, clusterin knockout; NOS 2, nitric oxide synthase 2; PT, proximal convoluted tubule; WT, wild type.

The distribution of the ARG 1^+^ and NOS 2^+^ cells mainly in the interstitial area and the injured TEC (Figure 6d) overlapped with that of F4/80 + Mφ (Figure 5a). At day 7 after IRI, the numbers of ARG 1^+^ (29.07 ± 5.84 cells hpf^−1^; Figure 6a) and NOS 2^+^ (14.52 ± 3.22 cells hpf^−1^; Figure 6b) in the WT group were significantly decreased compared with those in the control (ARG 1^+^, 85.99 ± 9.69 cells hpf^−1^, *P* = 0.0010; NOS 2^+^, 55.38 ± 9.42 cells hpf^−1^, *P* = 0.0021; *n* = 3).

Furthermore, the NOS 2^+^/ARG 1^+^ ratio was lower in the WT group (0.50 ± 0.02) than in the control (0.64 ± 0.06, *P* = 0.0179, *n* = 3, Figure 6c). These data confirmed the results obtained by FACS analysis on the ratios of CD80^+^/CD206^+^_,_ CD86^+^/CD206^+^, CD80^+^/CD163^+^ and CD86^+^/CD163^+^ at day 7 (Figure 4). Taken together, these suggested that there were significantly fewer M1 Mφ in the repaired WT kidneys at day 7 after IRI than in the poorly repaired control kidneys.

### CLU is associated with decreased polarization to M1 after lipopolysaccharide stimulation and increased phagocytosis

**Figure 7:**
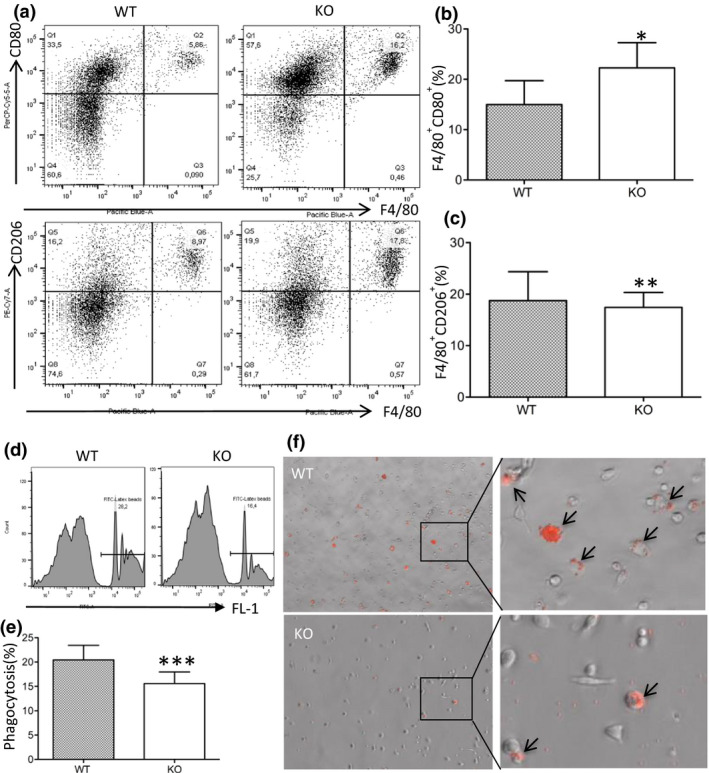
CLU is associated with decreased M1 polarization and increased phagocytosis in response to LPS stimulation. Peritoneal macrophages were harvested from WT and CLU‐KO mice after 3 days of intraperitoneal injection of LPS. M1 (F4/80^+^CD80^+^) and M2 (F4/80^+^/CD206^+^) phenotypes were determined by FACS analysis. **(a)** Representative histogram of three independent experiments. **(b)** The numbers of F4/80^+^CD80^+^ macrophages in the WT and the CLU‐KO control group. *P = 0.0054 (WT *versus* KO). **(c)** The numbers of F4/80^+^/CD206^+^ macrophages in the WT and the CLU‐KO control group. **P = 0.7754 (WT *versus* KO). **(d)** A representative histogram of phagocytosis of three independent experiments. **(e)** Phagocytosis of peritoneal macrophages. ***P = 0.006 (WT *versus* KO). **(f)** Representative microscopy images of the phagocytosis obtained by fluorescence microscopy from three independent experiments. Arrows indicate engulfed fluorescent (red) latex beads. Data in **b** (*n* = 5 per group), **c** (*n* = 5 per group) and **e** (*n* = 6 per group) are representative of three independent experiments and presented as the mean ± s.d. The difference between groups were analyzed by using the two‐tailed *t*‐test. CLU, clusterin; LPS, lipopolysaccharide; KO, clusterin knockout; WT, wild type.

To further reveal the effect of CLU on Mφ polarization in the spectrum of Mφ activation, the numbers of M1 (CD80^+^F4/80^+^) and M2 (CD206^+^F4/80^+^) phenotypes of peritoneal Mφ after stimulation by lipopolysaccharide (LPS) were determined by FACS analysis. Figure 7a shows a typical histogram of CD80^+^F4/80^+^ (M1) and CD206^+^F4/80^+^ (M2) in each group, and their quantification is illustrated in Figure 7b, c. The percentages of CD80^+^F4/80^+^Mφ (M1) in the WT mice (15.98 ± 10.65%) were significantly less than those in the CLU‐KO mice (22.26 ± 11.25%; *P* = 0.0054, *n* = 5). However, the difference between those of F4/80^+^CD206^+^ Mφ (M2) in the two groups were nonsignificant (WT, 18.76 ± 12.55%; KO, 17.43 ± 6.49%, *P* = 0.7754, *n* = 5). This suggested that CLU expression in Mφ suppressed polarization to the M1 phenotype, but not to the M2 phenotype, upon stimulation with LPS.

Phagocytosis is a key biological function of Mφ in adaptive immune response, tissue remodeling, wound healing and tissue homeostasis.^29^, ^30^ Therefore, we determined the effect of CLU on Mφ phagocytic activity by FACS analysis. As shown in Figure 7d, e, the levels of intracellular beads were significantly higher in the WT Mφ (20.45 ± 7.32%) than in the control (15.57 ± 5.89%, *P* = 0.006, *n* = 6). Fluorescence microscopy also confirmed that CLU enhanced Mφ phagocytosis (Figure 7f).

**Figure 8:**
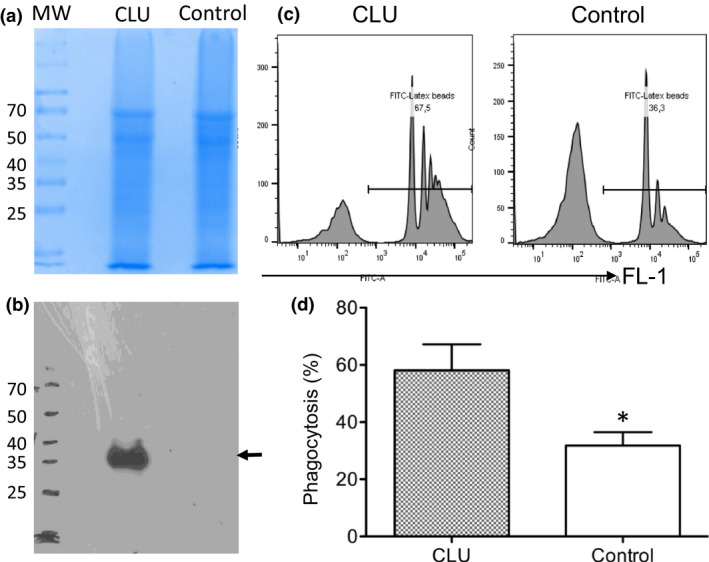
Addition of sCLU protein in the culture medium enhanced macrophage phagocytosis. RAW264.7 cells were incubated in the culture supernatant of the TEC‐CLU^hCLU^ (sCLU‐enriched medium) and TEC‐CLU^−/−^ (CLU null control) cells for 2 h, and cell phagocytosis was measured as described in Figure 7. **(a)** The protein fractions of the sCLU‐enriched (CLU) and the control media (Control) in 10% sodium dodecyl sulfate–polyacrylamide gel electrophoresis after Coomassie Blue staining. **(b)** The presence of sCLU protein as indicated by the arrow in the sCLU‐enriched medium using the western blotting. **(c)** Representative histogram of RAW264.7 cell phagocytosis in the presence or absence of sCLU. **(d)** The numbers of RAW264.7 cells containing the fluorescent beads. *P = 0.0417 (CLU *versus* control, two‐tailed *t*‐test). Data in **a**, **b** and **c** are representative of three independent experiments. Data in **d** are representative of three independent experiments and presented as the means ± s.d. (*n* = 4). CLU, clusterin; sCLU, secreted CLU; TEC, tubular epithelial cell.

CLU proteins are not only found inside the cells but are also secreted extracellularly.^31^ Thus, we examined the effect of extracellular CLU from cultured TEC‐CLU^hCLU^ cells on Mφ phagocytosis using RAW264.7 cells. The presence of CLU proteins in the culture supernatant after 24‐h incubation of the confluent TEC‐CLU^hCLU^ with K1 complete medium was confirmed using western blotting (Figure 8a, b). The effect of the exogenous secreted CLU from TEC‐CLU^hCLU^ cells compared with the supernatant (CLU null) from the TEC‐CLU^−/−^ cells on the phagocytosis of RAW264.7 cells was examined by FACS analysis. As shown in Figure 8c, d, the exogenous secreted CLU proteins significantly enhanced the phagocytosis of Mφ (58.125 ± 18.117) compared with the CLU‐negative control supernatants (31.825 ± 9.342, *P* = 0.0417, *n* = 4), suggesting that CLU stimulated the phagocytosis of Mφ.

## DISCUSSION

CLU proteins are commonly detected in physiological fluids, such as blood and semen, but it is also upregulated in stress‐induced cells, such as various tumor cells and renal TEC.^32^, ^33^ In addition, CLU expression in Mφ was weak or undetectable in the immunohistochemistry of lung tissue,^34^ but was upregulated in mature Mφ in human breast tumor tissues and the RAW264.7 Mφ cell line.^35^ In this study, CLU proteins were detected in THP1 human monocytes, RAW264.7 Mφ and peritoneal Mφ isolated from WT mice, and their expression was upregulated in THP1 cells and RAW264.7 cells following hypoxia. More importantly, we found that the CLU protein was a novel modulator for M1/M2 polarization in the model of kidney IRI and its repair.

Renal IRI can occur during trauma, vascular and cardiac surgery, embolic disease, iatrogenic injury, renal artery stenosis, circulatory arrest with resuscitation, partial nephrectomy and kidney transplantation.^6^ Its progression extensively overlaps with the remarkable ability to gradually repair itself through intrinsic and extrinsic mechanisms.^36^ During the repair process, phagocytes, such as Mφ, act as important scavengers of apoptotic cells or necrotic debris, and their presence in the kidney following IRI could alternatively represent a repair process.^37^ However, how Mφ participate in kidney damage, repair and fibrosis has not yet been fully understood.

In this study, we showed that upon the induction of renal IRI, the kidney tubules of WT mice showed significant damage at day 1, which peaked at day 3, and by day 7, exhibited decreased tubular injury, indicating tissue repair. By contrast, in the CLU‐KO group, tubular damage in the kidneys progressed and was not repaired by day 7,^27^ thus suggesting that the upregulation of CLU in the kidney mediates or is required for the renal repair or regeneration after IRI. However, the mechanisms underlying CLU‐mediated tissue repair are unclear. CLU is an anticell death protein that protects TEC from cell apoptosis mediated by inflammatory cytokines (e.g. tumor necrosis factor‐α and interferon‐γ) and hypoxia.^20^, ^38^ CLU in the TEC also regulates cell‐cycle progression and DNA repair, resulting in the promotion of TEC proliferation.^27^ All these functions of CLU in the TEC may contribute to the tubular repair we have observed. Furthermore, M2 Mφ and other anti‐inflammatory mediators may promote the intrinsic regeneration of remnant tubules to replace at least some of the necrotic cells.^39^, ^40^

Only a few tissue‐resident Mφ are present in naïve kidneys. However, following acute kidney injury, the monocytes were effectively recruited to the injured kidney to differentiate into Mφ, and the number of M1 Mφ peaked at day 3 and declined afterward.^7^, ^41^, ^42^ Meanwhile, the M2 Mφ population peaked at day 7 and gradually decreased.^7^, ^41^ However, whether the increase and the phenotype change in renal Mφ following IRI are due to the proliferation and polarization of resident Mφ are unknown.

During the early injury phase (days 1–3), the M1/M2 ratios were different when the different M2 markers (CD163 *versus* CD206) were used, but they were not significantly different between the WT and CLU‐KO groups (Figure 4). During the recovery phase (day 7), the M1/M2 ratios were consistently lower in the WT group than in the control, regardless of which M2 marker was used (Figure 4). A lower M1/M2 Mφ population was correlated with renal repair in the WT kidneys, which was not observed in the CLU‐KO control (Figure 1), suggesting an unknown role of CLU in Mφ differentiation to functional phenotypes (M1 and/or M2), particularly at the late stage of tissue repair after IRI. Furthermore, the fewer Mφ in the WT group than in the control may be due to the inhibitory activity of CLU we found in cultured TECs.^38^, ^43^ By inhibiting cell migration, the infiltration of WT monocytes to the damaged kidneys may occur more slowly than that of CLU‐KO monocytes, which needs further investigation.

Mφ from the peritoneal cavity of mice are commonly used for functional studies.^44^ When these peritoneal Mφ from the WT group were stimulated by a low dose of LPS, the F4/80^+^CD80^+^ M1 Mφ significantly decreased compared with the CLU‐KO mice, but there was no difference in the F4/80^+^CD206^+^ M2 Mφ between the two groups (Figure 7). This finding indicated that CLU negatively regulates Mφ polarization to the proinflammatory or “killing” M1 phenotype, which is consistent with the lower ratios of M1/M2 Mφ in the kidneys of the WT group during the recovery phase.

In addition, the NOS 2^+^ (M1 marker) and ARG 1^+^ (M2 marker) cells were higher than the F4/80^+^ (Mφ marker) cells in the renal sections (Figure 6). The additional positive staining of NOS 2 may be due to the expression of NOS 2 in the TECs, the levels of which can be upregulated by the stimulation with Type 1 helper proinflammatory cytokines.^45^, ^46^ ARG 1 is not normally expressed in the kidneys of normal or diabetic mice,^47^, ^48^ and in Mφ, ARG 1 expression is induced by Type 2 helper cytokines, such as IL‐4 and IL‐13.^49^, ^50^ Whether ARG 1 is induced by Type 2 helper cytokines in any of the kidney cells, such as TEC, at the recovery phase after IRI remains unknown. Furthermore, because different antibodies were used for detecting these marker proteins, the differences in the sensitivity and specificity among these antibodies may have also contributed to the observed discrepancy in the numbers of Mφ.

There may be several mechanisms by which the anti‐inflammatory M2 Mφ promote tubular repair during the recovery phase. Mφ‐derived Wnt7b, BRP‐39 and IL‐22 directly stimulate tubular cell proliferation,^16^ whereas Mφ phagocytosis clears damaged tissues for tissue regeneration and angiogenesis.^29^ A previous study has reported that CLU promotes Mφ phagocytosis in the clearance of late apoptotic cells.^51^ Here, we have showed that the phagocytic activity of the CLU‐expressing WT Mφ was higher than that of the CLU‐KO control (Figure 7), and that the addition of secreted CLU protein produced by CLU‐expressing TEC‐CLU^hCLU^ significantly enhanced the Mφ phagocytosis (Figure 8). Previous studies also have confirmed that the phagocytic activity of M2 Mφ is more efficient than that of M1 cells.^52^, ^53^ They attributed this to the higher activity of M2 Mφ in scavenging debris and tissue remodeling.^54^, ^55^ Furthermore, following hypoxia incubation of RAW264.7 cells for 24 h, CLU expression was enhanced (Figure 3), but those of the M1 marker CD80 and the M2 marker CD206 decreased, while the phagocytosis was also increased (data not shown). These observations may imply that the upregulated and endogenous CLU expression and/or the added exogenous CLU protein could enhance the phagocytic capacity of Mφ regardless of its phenotype. These studies suggest that CLU‐mediated Mφ phagocytosis contributes at least partly to tissue repair at day 7 after IRI in the WT mice.

In conclusion, Mφ play critical roles in renal inflammation, renal tissue damage and repair and fibrosis.^7^ Our previous studies have reported that the CLU‐expressing WT kidneys are more resistant to IRI than the control CLU‐KO kidneys^20^ and exhibit tissue repair at day 7 following IRI.^27^ We have also revealed that CLU expression reduces Mφ population and tissue fibrosis in the kidney after 30 days of IRI.^25^ To the best of our knowledge, this is the first report to demonstrate that CLU expression in Mφ is upregulated in hypoxia. CLU reduced the numbers of Mφ, and its abundance correlated with less severe kidney damage following IRI. Furthermore, we revealed that CLU specifically facilitates M1 polarization and enhances Mφ phagocytic activity. However, a further understanding of the molecular mechanisms underlying the regulation of Mφ infiltration, proliferation, activation (polarization to M1/M2 phenotypes) and phagocytic activity by CLU is warranted, which may lead to the development of an effective CLU‐based therapeutic strategy for kidney tissue damage and kidney disease progression.

## METHODS

### Animals and cells

Male 8‐ to 12‐week‐old WT (C57BL/6, B6) and CLU‐KO mice in B6 background (B6‐CLU^−/−^) were obtained from the breeding colonies in the animal facility at the Jack Bell Research Centre (Vancouver, BC, Canada). The animals were littermates and cohoused in the same room of the facility. All animal procedures were in accordance with the Canadian Council on Animal Care, and the study protocol (A16‐0271) was approved by the Animal Use Subcommittee of the University of British Columbia (Vancouver, BC, Canada).

The human monocyte THP‐1 cells (ATCC TIB‐202) and mouse Mφ RAW264.7 cells (ATCC TIB‐71) were purchased from the American Type Culture Collection (Old Town Manassas, VA, USA) and were grown in Roswell Park Memorial Institute‐1640 medium containing 5% of fetal bovine serum (FBS). The CLU‐expressing TEC (herein, TEC‐CLU^hCLU^) were established from the renal TEC of CLU‐KO mice through the stable expression of human CLU isoform 1 cDNA using the pHEX6300 vector as described previously,^43^ whereas CLU null TECs that were transfected with the empty expression vector pHEX6300 served as the CLU‐negative control cells (TEC‐CLU^−/−^) for TEC‐CLU^hCLU^. Both TEC cell types were grown and maintained in a complete K1 medium.^56^ The cell cultures were expanded and incubated at 37°C in 5% CO_2_ atmosphere.

### Generation of the renal IRI model

Unilateral IRI was induced in the left kidneys of WT and KO mice according to a routine surgical procedure in our laboratory.^25^, ^27^ Briefly, the mice were anesthetized by an intraperitoneal injection of ketamine (100 mg kg^−1^) and xylazine (10 mg kg^−1^), and by inhalation of isoflurane as needed. The kidneys were exposed through a flank incision, and ischemia was induced by clamping the left renal pedicles at 32°C for 30 min. After the clamps were released, the reperfusion of the kidneys (normal red again) was visually confirmed.

To examine the tissue injury and cellular infiltration of the left kidney at days 1, 3 and 7 after IRI, the nonischemic right kidney was kept in order to reduce animal suffering. To determine the kidney function after IRI, the nonischemic right kidney was removed, and blood samples were collected from the tail vein using a lithium heparin tube before surgery (day 0) and at days 3 and 7 after IRI.

### Determination of serum creatinine and blood urea nitrogen levels

The levels of serum creatinine and blood urea nitrogen were analyzed by the Clinical Chemistry Laboratory at the Vancouver Coastal Health Regional Laboratory Medicine (Vancouver, BC, Canada) using the Dimension Vista System (Siemens Healthcare Diagnostics Inc., Newark, DE, USA).

### Histopathological examination of tissue injury

The kidneys of WT and KO mice after IRI were randomly selected for histological analyses of renal tubular damage as described previously.^25^ Briefly, a coronal tissue slice (4‐μm thick) was cut through the midportion of each kidney, fixed in 10% neutral buffered formalin, embedded in paraffin and stained with hematoxylin and eosin. The tissue sections were visualized using a Leica SCN400 Slide scanner, followed by blind examination using the Digital Image Hub—a SlidePath software (Leica Microsystems Inc., Concord, ON, Canada).

The number of damaged tubules, including intraductal cast formation, atrophy (cell loss), tubular cell flattening or vacuolation, was expressed as the percentage of the total number of tubules in each view of the renal cortical region under 400× magnification. The average percentage of at least 20 randomly selected, non‐overlapping views represented the number of injured tubules in each kidney.

### Isolation of leukocytes from kidneys and spleens

Leukocyte isolation from the kidneys or spleens was performed as described previously with minor modifications.^57^, ^58^ The WT and KO mice were randomly selected from each group for each time point. Briefly, after perfusion with cold phosphate‐buffered saline (PBS), the kidney or spleen tissues were minced and gently crushed and filtered using a 40‐μm cell strainer (BD Biosciences, Mississauga, ON, Canada). The tissues were washed with PBS, and the erythrocytes were removed through incubation with lysis buffer (containing 0.15 m NH_4_Cl, 1.0 mm KHCO_3_, 0.1 mm EDTA; pH 6.8) for 5 min. After washing with PBS, the purified kidney‐infiltrating cells or splenocytes were finally suspended in PBS (with 1% FBS) for phenotype determination.

### Fluorescence‐activated cell sorting analysis

The phenotypes and the percentages of Mφ were determined by FACS analysis with fluorescence dye‐conjugated antibodies as described previously.^25^ The cell suspensions (10^5^ cells/sample in PBS with 1% FBS) were incubated in the dark for 30 min at 25°C with the following antibodies: rat monoclonal anti‐F4/80 (clone BM8, Thermo Fisher Scientific, Waltham, MA, USA) for the primary gating of Mφ; rat monoclonal anti‐CD80 (clone 16‐10A1, Thermo Fisher Scientific) for F4/80^+^CD80^+^ M1 Mφ or anti‐CD86 (clone GL1, Thermo Fisher Scientific) for F4/80^+^CD86^+^ M1 Mφ and anti‐CD206 (clone MR6F3, Thermo Fisher Scientific) for F4/80^+^CD206^+^ M2 Mφ or anti‐CD163 (clone TNKUPJ, Thermo Fisher Scientific) for F4/80^+^CD163^+^ M2 Mφ.

The percentages of Mφ were determined through positive staining with anti‐F4/80, anti‐MAC3 (clone M3‐84, BD Biosciences) or anti‐CD11b (clone M1‐70‐15, BD Biosciences). Briefly, after incubation, the cells were washed with PBS, and the positively stained cells were identified using a FACSCanto II cell analyzer (BD Biosciences) and quantified using the FlowJo software (Tree Star Inc., Ashland, OR, USA).

### Immunohistochemistry

The kidneys were perfused with PBS, followed by formalin fixation, paraffin embedding and sectioning as described above. The levels of F4/80, ARG 1 and NOS 2 proteins were assessed. In brief, after deparaffinization and rehydration, the buffered formalin‐fixed tissue sections were treated with 3% H_2_O_2_ in Tris‐buffered saline (pH 7.4) for 30 min at room temperature to quench the endogenous peroxidase. This was followed by permeabilization with 0.2% Triton X‐100 for 10 min, washing with Tris‐buffered saline containing 0.1% Tween‐20 and blocking with 5% normal goat serum. Then, the sections were incubated overnight at 4°C with the following secondary antibodies (1:50 dilution): primary rat monoclonal anti‐F4/80 (Thermo Fisher Scientific), rabbit anti‐ARG 1 (clone PA5‐85267, Thermo Fisher Scientific) or rabbit anti‐NOS 2 (Santa Cruz Biotechnology, Inc., Santa Cruz, CA, USA). The immune complexes of F4/80, ARG 1 or NOS 2 were detected using a Vector M.O.M. Immunodetection kit following the manufacturer’s protocol (Vector Labs, Burlington, ON, Canada) and were visualized using a 3,3′‐diaminobenzidine peroxidase substrate. The numbers of F4/80^+^, ARG 1^+^ or NOS 2^+^ in each tissue section were counted at 400× magnification in a blinded manner. The negative control was incubated with normal rat or rabbit IgG instead of primary antibodies. The nuclei were counterstained with hematoxylin.

### Western blot analysis

CLU protein levels in the protein extracts of human THP1 monocytes, mouse RAW264.7 and primary Mφ, and the protein precipitation of culture supernatants were determined using the western blot as described previously.^20^, ^43^ Briefly, the samples were fractionated using 10% sodium dodecyl sulfate–polyacrylamide gel electrophoresis and transferred onto nitrocellulose membranes. The CLU proteins on the blots were identified using the polyclonal goat anti‐CLU antibody (Santa Cruz Biotechnology), and the protein–antibody bands were visualized using donkey anti‐goat IgG H&L‐Alexa Fluor 680 (Abcam Canada Toronto, ON, Canada) on an Odyssey LI‐COR imaging system (LI‐COR, Inc., Lincoln, NE, USA). To confirm the loaded proteins in each sample, the levels of β‐actin on the same blot were probed using anti‐β‐actin antibody (Sigma‐Aldrich Canada, Oakville, ON, Canada), or the same protein fractions in sodium dodecyl sulfate–polyacrylamide gel electrophoresis were stained using 0.25% Coomassie Blue R‐250 (Sigma‐Aldrich Canada).

### Cell isolation from mouse peritoneal cavity

To investigate the effect of CLU on the polarization of Mφ in response to inflammation, 10 μg of LPS (Sigma‐Aldrich Canada) in 200 μL of PBS was intraperitoneally injected to WT or CLU‐KO mice.^44^ After 3 days, the Mφ were isolated from the mouse peritoneal cavity and purified. Briefly, the mice were euthanized, and the outer layer of the peritoneum was incised carefully after disinfection, and ice‐cold PBS (contain 3% FBS) was subsequently injected into the peritoneal cavity. The cells from the peritoneum were dislodged by gentle massage into the PBS. After a 5‐min massage, the fluid from the peritoneum was collected into a tube. Subsequently, repeated lavage of the abdominal cavity with PBS was performed, and the fluid was collected in the same tube. The cells were pelleted by centrifuging at 980 *g* at 4°C for 5 min and were resuspended in PBS. The number of viable cells was counted using a TC10 automated cell counter (Bio‐Rad Laboratories Canada, Mississauga, ON, Canada).

### Phagocytosis assay

The primary Mφ were seeded in Roswell Park Memorial Institute‐1640 medium containing 5% of FBS, whereas the RAW264.7 Mφ were seeded in the culture supernatant of TEC‐CLU^hCLU^ or TEC‐CLU^−/−^ cells incubated in 5% CO_2_ atmosphere at 37°C. For the primary Mφ from the peritoneal cavity, the cells were seeded at a density of 1 × 10^6^ cells/well in 24‐well plates and cultured for 2 h, followed by the removal of nonadherent cells by gentle washing with PBS three times. For the RAW264.7 Mφ, the cells were incubated with the TEC culture supernatant at a density of 1 × 10^6^ cells/well in 24‐well plates for 2 h. In both the primary and RAW264.7 Mφ cultures, 2 μL of fluorescent latex beads (Sigma‐Aldrich Canada) was added per well for 2 h. Phagocytosis was determined by counting the number of cells engulfing the beads by FACS as described above.

### Statistical analysis

Data are presented as the mean ± standard deviation (s.d.) or the standard error of the mean (s.e.m.), and ANOVA or two‐tailed *t*‐tests were used for comparisons between groups as indicated in the figure captions. All statistical analyses were performed using the GraphPad Prism 7 (GraphPad Software, Inc., La Jolla, CA, USA). A *P*‐value ≤ 0.05 was considered significant.

## CONFLICT OF INTEREST

The authors declare that they have no competing interests.

## Author Contribution

**Xiaodong Weng:** Conceptualization; Data curation; Formal analysis; Funding acquisition; Investigation; Methodology; Writing‐original draft; Writing‐review & editing. **Haimei Zhao:** Data curation; Investigation; Methodology; Writing‐review & editing. **Qiunong Guan:** Data curation; Formal analysis; Investigation; Methodology; Project administration; Writing‐review & editing. **Ganggang Shi:** Data curation; Formal analysis; Investigation; Methodology; Writing‐review & editing. **Shijian Feng:** Data curation; Formal analysis; Investigation; Methodology; Writing‐review & editing. **Martin E Gleave:** Conceptualization; Resources; Software; Visualization; Writing‐review & editing. **Christopher CY Nguan:** Conceptualization; Project administration; Resources; Software; Supervision; Visualization; Writing‐review & editing. **Caigan Du:** Conceptualization; Data curation; Formal analysis; Funding acquisition; Project administration; Resources; Software; Supervision; Validation; Visualization; Writing‐original draft; Writing‐review & editing.
